# Childhood Absence Epilepsy Associated With Concomitant Centrotemporal Spikes

**DOI:** 10.7759/cureus.28489

**Published:** 2022-08-27

**Authors:** Bosanka Jocic-Jakubi, Darina Jocic, Rajesh P Poothrikovil, Amna Al-Futaisi

**Affiliations:** 1 Pediatric Neurology, Sultan Qaboos University Hospital, Muscat, OMN; 2 Pediatrics, Clinical Center of Niš, Niš, SRB; 3 Clinical Neurophysiology, Sultan Qaboos University Hospital, Muscat, OMN; 4 Pediatrics, Sultan Qaboos University, Muscat, OMN

**Keywords:** eeg, bects, rolandic epilepsy, cae, absence seizures

## Abstract

Objectives

The coexistence of generalized epileptiform discharges of 3Hz spike-and-wave complexes, which are the hallmark of childhood absence epilepsy (CAE), and centrotemporal spikes, which are characteristic of benign epilepsy with centrotemporal spikes (BECTs) in the same or subsequent EEGs appears to be very rare. Only a few published reports have shown a possible concomitance of CAE and BECTs electrographic changes. The study aimed to analyze electrographic and clinical features of patients with CAE who had concomitant or subsequent EEG features of BECTs.

Method

During a five-year analysis period (2014-2018), 277 children with BECTs and 93 children with CAE were diagnosed and treated at the pediatric neurology unit of Sultan Qaboos University Hospital (SQUH) in Muscat, Oman. Nine patients were identified to have overlapping EEG findings of both epileptic syndromes. We then analyzed the nine children's clinical features, outcomes, and EEG findings in detail.

Results

The clinical onset of all our patients aged 5-14 years (six boys, three girls) was characterized by the absence of seizures, either typical (seven children) or atypical (two children). Six out of nine patients presented with concomitant electrographic features of both syndromes, whereas three patients experienced the EEG pattern of two syndromes at different times. All nine children were treated with valproate as the first-line medication, with reasonable seizure control. However, three patients required a second add-on medication. Despite good seizure control, six of our patients had poor school performance and five children had comorbid conditions such as ADHD and learning disability.

Conclusion

The coexistence of CAE and BECTS is described in the literature albeit rare. This overlap is mostly in electrographic features with or without the clinical features seen in both syndromes.

## Introduction

Introduction

Childhood absence epilepsy (CAE) and benign epilepsy with centrotemporal spikes (BECTS) are common epilepsy syndromes in children. The two syndromes share some common features such as the age of onset and an overall good prognosis. However, they have very different pathophysiologies. Some studies have described the coexistence of CAE and BECTS in the same patient. Nonetheless, this phenomenon is to date scarcely reported in the literature with only a few published articles showing a possible concomitance of clinical or EEG features of CAE and BECTs [1-7]. Furthermore, the coexistence of generalized epileptiform discharges (GEDs) 3Hz/sec spike-and-wave complexes which are the hallmark of absence epilepsy (CAE), and epilepsy with centrotemporal spikes (BECTs) in the same or different EEGs appears to be very rare but this combination of conditions nonetheless exists.

Several theories have been postulated to explain this coexistence, including the possibility of aggravation by antiseizure medications (ASM) such as carbamazepine and phenobarbitone and conversion to absence with BECTS as secondary bilateral synchrony rather than a real association [4,8,9]. Six patients with BECTS were described to develop absences and generalized spike-wave discharges when on treatment with carbamazepine [4]. It was concluded that antiepileptic drug, namely carbamazepine was the reason for the occurrence of clinical absences or "absence-like" generalized paroxysms in children with BECTS. This conclusion was based on the fact that this conversion developed one or three months after ASM treatment was introduced and not with the onset of epilepsy. Another study reported a child with BECTS on valproate who developed absence seizures when phenobarbitone was added to improve seizure control, which disappeared immediately after phenobarbitone was withdrawn [9].

Several authors have reported children with consecutive clinical semiology of those two epileptic syndromes [5,10]. Gambardella et al. (1996) described the later occurrence of CAE in three patients with a history of BECTS [5], while Jun et al. (2019) published a case of an eight-year-old girl with typical Rolandic seizure, who developed frequent brief episodes of staring and unresponsiveness during the daytime [10]. In a systematic record review from eight epilepsy centers, 11 children were identified with the electro-clinical concomitant occurrence of CAE and BECTS. Four out of 11 patients presented with concurrent features of both syndromes, whereas the remaining seven experienced the two syndromes at different times [11].

The aim of the study was to analyze patients with BECTS or absence epilepsy who developed simultaneous or subsequent clinical and or electrographic features of each epileptic syndrome and discuss their clinical features, response to treatment, and developmental outcome. 

## Materials and methods

During the five-year study period (January 2014-2018), 4492 pediatric patients have been diagnosed and treated at the pediatric neurology unit and neurophysiology laboratory, Sultan Qaboos University Hospital (SQUH), Muscat, Oman. Among them, 277 were found to have centrotemporal (Rolandic) discharges and 93 patients showed 3Hz/sec generalized spike and waves (GSW) discharges on EEG. A retrospective analysis was carried out to identify patients with either syndrome who had overlapping clinical or electrographic features. Clinical information including age, gender, seizure type and frequency, neurological examination, neuroimaging, comorbidity, antiepileptic medication, seizure control, and school performance were obtained from the SQUH hospital information system and were retrospectively analyzed. All EEG data were obtained from the EEG database at SQUH and all the positive EEGs were reviewed.

EEGs were recorded with electrode placement according to the International 10-20 system. Hyperventilation for five minutes and intermittent photic stimulation with flash frequencies starting at 2 Hz and increasing by 3 Hz intervals up to 30 Hz were performed on all patients at each recording. The seizure type was classified according to the 2017 International League Against Epilepsy Classification as CAE or BECTS [12].

## Results

Among the 370 patients who were identified to have either BECTs (277) or CAE (93), nine of them (2.4%) were found to have overlapping EEG findings seen in the two childhood epilepsy syndromes. A detailed review of those nine patients was carried out. All our patients, six boys and three girls, aged 5-14 years demonstrated clinical features consistent with childhood absence epilepsy, either typical (seven children) or atypical absences (two children). Five children had staring associated with lip-smacking, and none of them experienced any clinical features of BECTs in the further course of the disease. MRI brain was performed on three of the nine patients. Age at seizure onset ranged between 3.5 to 8 years of age (mean 5.3 years). Six out of nine (67%) patients presented with concomitant electrographic features of both syndromes, whereas three patients (33%) experienced the EEG pattern of the two syndromes at different times, in two cases one year after, and in one case two years after the onset of absences. The follow-up period ranged from 1.5 years to seven years (mean five years) and in four of our patients (44%) EEG was normal on the last follow-up (Table 1).

**Table 1:** Outcome of children with absence epilepsy and BECTs trait ADHD: attention deficit/hyperactivity disorder, Pos: positive, Neg: negative, VPA: valproate, LTG: lamotrigine, LEV: levetiracetam, CZP: clonazepam, CLB: clobazam, ND: not done, N: normal, AS: absence seizures; BECTs: benign epilepsy with centrotemporal spikes

Patients	Initial treatment	Additional treatment	Treatment on last F/U	Seizure control on last F/U	School performance	IQ	Comorbidities
1	VPA	No	VPA	Yes	Average	ND	No
2	VPA	Atomoxetin, LEV	Atomoxetin, LEV, VPA	Yes	Poor	82	ADHD
3	VPA	No	No	Yes (5y)	Poor	114	ADHD
4	VPA	CZP, LTG, CLB	VPA+CLB	Yes (2y)	Poor	98	ADHD
5	VPA	LTG	VPA	No	Poor	94	No
6	VPA	No	VPA	Yes (3y)	Poor	78	Learning dis.
7	VPA	LTG	VPA+LTG	No	Average	ND	No
8	VPA	No	VPA	Yes	Still good	ND	No
9	VPA	Methylphenidate	VPA	Yes	Poor	93	ADHD + Learning dis

**Figure 1:**
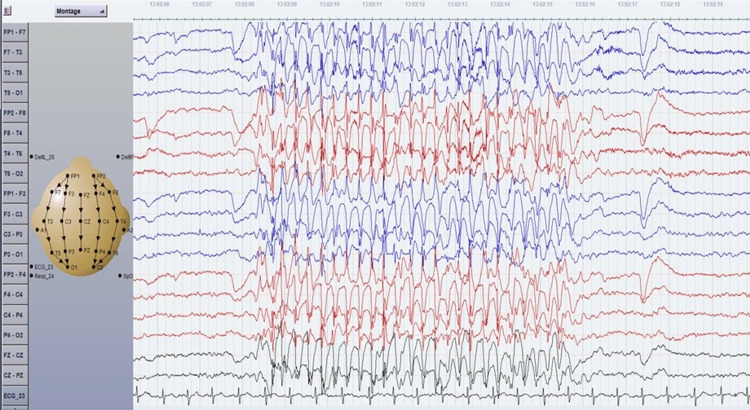
EEG of a bipolar montage of an eight-year-old boy with absence seizures since four years of age and concurrent overlapping of GEDs (3Hz) and benign Rolandic trait. Ictal EEG showed spontaneous absence seizures. GED: generalized epileptiform discharge

**Figure 2:**
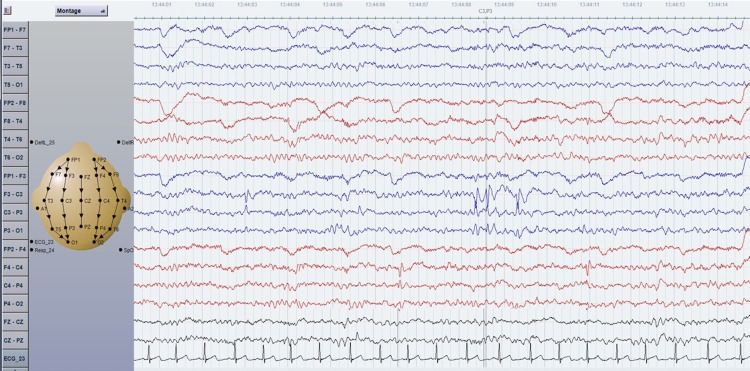
EEG of a bipolar montage of an eight-year-old boy with absence seizures at four years of age and concurrent overlapping of GEDs (3Hz) and benign Rolandic trait. One year after experiencing absence seizures, his interictal EEG showed independent left and right centrotemporal spikes.

**Figure 3:**
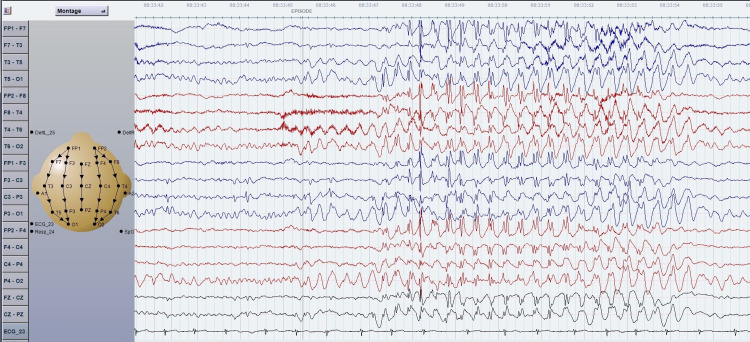
EEG of a bipolar montage of a six-year-old boy with concomitant absence seizures and BECTs trait. The EEG showed focal epileptiform discharges over the left centro-parietal region and generalized 3Hz spike-and-wave complexes, associated clinically with staring and eye blinking. BECTs: benign epilepsy with centrotemporal spikes

In all nine patients, the initial treatment was valproate as ethosuximide was not available at our hospital. There was good seizure control in six patients, however, in three of them, lamotrigine or benzodiazepines were added. Despite good seizure control, these six patients (67%) had poor school performance early in the course of their presentation, and in five of them, (55%) comorbidities such as ADHD and learning disability were found. The neurologic examination was normal in all patients. An IQ test was done months after starting treatment and revealed a below-average IQ in two of our patients, on the lower side of average in three patients, a high score in one (IQ 114), and three were not tested. A positive family history of epilepsy was seen in three patients (33%); all of them were from the maternal side and had two relatives diagnosed with absence epilepsy. An MRI brain scan was conducted on three patients and showed no abnormalities. Two patients expressed a generalized photoparoxysmal response (PPR) during intermittent photic stimulation (Table 2). EEG patterns of our patients were presented in (Figures 1-3) to show examples of concurrent and subsequent EEG traits overlap of CAE and BECTs in the same patients.

**Table 2:** Electroclinical characteristics of absence epilepsy and BECTS trait Abs: absence, Sz: seizure, CT sp: centrotemporal spikes, PPR: photoparoxysmal response, CAE: childhood absence epilepsy, Abn: abnormal, N: normal, GEDs: generalized epileptiform discharges, ESES: electrical status epilepticus, BECTs: benign epilepsy with centrotemporal spikes

Patients	Age at onset (years)	Gender	Initial seizure type	Initial EEG awake	Initial EEG sleep	Overlap CAE and benign Rolandic trait	Last F/U EEG	F/U (y)
1	4	F	Abs	3Hz/s	CT sp	Consecutive one year after	CT sp	5
2	8	M	Abs	3Hz/s + CT sp	GEDs + CT sp	Concurrent	N	6
3	5	M	Abs	3Hz/s + CT sp	CT spikes	Concurrent	3Hz/s + CT sp	7
4	7	M	Abs	3Hz/s + CT sp	ESES	Concurrent	N after 10 mos	3
5	6	M	Abs	3Hz/s	CT sp	Consecutive two years after	CT sp	4
6	4	M	Abs	3Hz/s + PPR	CT sp	Consecutive one year after	N after 4 y	5
7	3.5	M	Abs	3Hz/s + CT sp	GEDs + CT sp	Concurrent	GEDs but no sz.	1.5
8	5	F	Abs	3Hz/s + CT sp	CT sp	Concurrent	N	1.5
9	6	F	Abs	3Hz/s + CT sp + PPR	Not done	Concurrent	GEDs	3

## Discussion

The coexistence of two idiopathic childhood epilepsy syndromes, namely CAE and BECTS is described but is very infrequently mentioned in the literature. This overlap is mostly in electrographic features with or without the clinical features seen in both syndromes. The underlying pathophysiological mechanism for this coexistence remains unknown and controversial. In our cohort, all of our patients presented with clinical absence seizures and were treated with valproate as first-line medication. Six of those patients had concurrent electrographic features of absence epilepsy and focal centrotemporal spikes. While three patients developed centrotemporal spikes one to two years after the onset of CAE, a study conducted by Ramelli et al. (1998) among 1114 children with epilepsy found 80 children with CAE and 42 with BECTS [6]. Five children (three girls and two boys) showed either concomitant or consecutive EEG characteristics for CAE and BECTS. In three patients, the primary type of seizure was characterized as focal motor with clinical and electrographic features that were consistent BECTs. A few months later they developed absence seizures. In another two children absences were the first manifestation of epilepsy and later on, on EEG centrotemporal spikes were noted but without any clinical seizures to suggest BECTs [2].

In the past four decades, it was shown that spike-wave discharges recorded in absences and other forms of generalized seizures are mediated through thalamic and thalamocortical circuits [13]. Many expressing increasing interest in the pathophysiology of generalized seizures postulated that absence seizures could originate from restricted regions of the cerebral cortex and selected cortical networks [14-16]. Huntsman et al. (1999) demonstrated that the thalamic circuit, responsible for the origin of 3Hz GEDs, could produce highly organized and repetitive activity, such as centrotemporal epileptiform discharges [17]. Although it is difficult to explain the association of two epileptic syndromes in the same patient, recent animal models suggested that BECTS and CAE could be genetically and pathophysiologically linked, which can be one of the explanations for the concomitant or consequent existence of CAE and BECTs [18-20]. The discussion of whether absence seizures should still be regarded as "generalized" seizures has been raised by the existence of clearly defined cortical networks or regions where hemodynamic and electrographic abnormalities are first noticed before spreading to other areas [21].

Anyanwu et al. (2012) reported an 11-year-old with typical CAE (clinically and electrographically) with the onset of seizures at the age of six years [1]. After ethosuximide introduction, she became seizure-free but her follow-up EEGs consistently showed right centrotemporal spikes not associated with focal clinical seizures. Another patient was described to have BECTS but later developed atypical absence seizures following treatment with phenobarbital. This patient continued to have both types (focal and generalized) of EEG abnormalities [9]. These authors attributed that this conversion is likely drug-induced by ethosuximide and phenobarbital, respectively.

The presence of additional comorbidities in patients with absence epilepsy and Rolandic traits was first described in by Datta et al. [3]. ADHD was seen in 24% of their 17 patients and difficulties in school performance in 65% (11 patients), while language disorder was found in 35%. In our study, ADHD and learning disability were seen in 55% and poor school performance was seen in 67% of our patients. Three of the patients had microarray-based comparative genomic hybridization testing which was negative. The occurrence of both epilepsy syndromes in the same patient usually doesn't appear to have a negative influence on the clinical outcome, but in our patients, the majority of them had learning difficulties which required the children to be placed in special education classes.

Three (33%) out of nine of our patients had a positive family history of epilepsy (two for absence epilepsy). A previous multicenter study described only two patients (18%) who had a positive family history of epilepsy (neither BECTS nor CAE) [22]. However, the numbers described in this report remain to be too small to make any firm conclusions. The findings of this study are subject to several limitations. The small number of subjects, although reflecting the rarity of this phenomenon, remains a major limitation. Another limitation is the retrospective nature of the study. The fact that not all of the participants underwent IQ testing further impacts the observation of increased cognitive impairment and educational challenges within the study group. Further collaborative prospective multicenter studies are needed to make accurate and generalizable conclusions about the coexistence of CAE and centrotemporal spikes of BECTS. Studies with a good sample size will also improve our knowledge of this association in order to improve the outcomes and the impacts of clinical versus electrographic concomitance.

## Conclusions

The rare coexistence of CAE and centrotemporal spikes of BECTS is shown in a retrospective analysis of this small series of patients with epilepsy. In our study group, the described cohort of children has an unfavorable long-term prognosis in terms of learning and school performance. The overlapping of Rolandic spikes and absence epilepsy either concurrently or consecutively may be a marker of additional cognitive disturbance. More rigorous neuropsychological testing in such children may be required to shed light on the impact of the presence of electrographic features of two clinically and electrographically distinct syndromes. Our study describes a small number of patients, which signifies the rarity of the phenomena. Larger multicenter studies are needed to confirm or dispute such associations and to make more meaningful conclusions.
